# Altered Serum MicroRNAs as Novel Diagnostic Biomarkers for Atypical Coronary Artery Disease

**DOI:** 10.1371/journal.pone.0107012

**Published:** 2014-09-08

**Authors:** Jun Wang, Yinghao Pei, Yong Zhong, Shisen Jiang, Jiaqing Shao, Jianbin Gong

**Affiliations:** 1 Department of Cardiology, Jinling Hospital, School of Medicine, Nanjing University/Nanjing General Hospital of Nanjing Military Command, Nanjing, Jiangsu Province, P.R. China; 2 Department of Health-Care, Jinling Hospital, School of Medicine, Nanjing University/Nanjing General Hospital of Nanjing Military Command, Nanjing, Jiangsu Province, P.R. China; 3 Department of Endocrinology, Jinling Hospital, School of Medicine, Nanjing University/Nanjing General Hospital of Nanjing Military Command, Nanjing, Jiangsu Province, P.R. China; Sun Yat-sen University, China

## Abstract

**Background and Aim:**

Atypical coronary artery disease (ACAD) is characterized by atypical angina pectoris or silent myocardial ischemia. However, conventional diagnostic techniques are insufficient to identify this subtype of coronary atherosclerotic pathology, and specific and sensitive markers for diagnosing ACAD are still currently lacking. The aim of the present study is to identify a novel serum microRNA (miRNA) expression profile of ACAD patients and evaluate its clinical diagnostic value.

**Patients and Methods:**

127 patients who were diagnosed with ACAD and 54 age-matched controls were enrolled in this study. An initial screen of miRNA expression was performed in serum samples from 35 patients and 20 controls using TaqMan Low Density Array. A stem-loop quantitative reverse-transcription PCR (RT-qPCR) assay was conducted in the training and validation sets to confirm the levels of the altered miRNAs in 122 patients with ACAD and 68 controls. In addition, the potential target genes of the altered miRNAs were predicted using bioinformatics methods.

**Results:**

The TaqMan low density array and RT-qPCR analysis identified four serum miRNAs including miR-487a, miR-502, miR-208 and miR-215 that were significantly increased, while one miRNA, miR-29b, that was significantly decreased in ACAD patients compared with normal controls (*P*<0.05). The area under the receiver-operating-characteristic (ROC) curve (AUC) for the combined 5 serum miRNAs were 0.850 (95% CI, 0.734–0.966, *P*<0.001) and 0.909 (95% CI, 0.858–0.960, *P*<0.001) for the training set and validation set, respectively. In addition, target gene prediction showed that these five altered miRNAs are involved in affecting various aspects of cardiac or vascular remodeling, especially in the pathway involving inflammation and fibrosis.

**Conclusion:**

Our findings indicate that the five altered serum miRNAs could be novel non-invasive biomarkers for ACAD and may also represent potential therapeutic targets for atherosclerosis and myocardial ischemia.

## Introduction

Atypical coronary artery disease (ACAD) is a type of peripheral arterial disease characterized as atypical angina pectoris or silent myocardial ischemia. Numerous studies have shown that the presence of ACAD predicts a much higher risk of major adverse cardiovascular events [1]. Because both the necessity and the benefit of treating ACAD remains in question, particularly in asymptomatic patients without a history of coronary artery disease (CAD), useful biomarkers for ACAD are needed [2], [3]. Currently available diagnostic techniques such as the use of traditional cardiac biomarkers or electrocardiogram are limited in their ability to identify patients who present with uncommon symptoms or silent myocardial ischemia in its early stages [3]. Therefore, identification of simple, specific and non-invasive biomarkers that can help diagnose ACAD in its early phase is urgently needed.

MicroRNAs (miRNAs) are a class of small non-coding RNAs approximately 22 nucleotides in length that regulate gene expression by accelerating mRNA degradation or inhibiting translation at the posttranscriptional level [4]. Numerous studies have revealed that miRNAs play a pivotal role in the control of most biological processes including cell differentiation, proliferation, migration, development and apoptosis. Importantly, there is increasing evidence that miRNAs are involved in various pathological processes including cardiovascular disease, angiogenesis and inflammation. For instance, some muscle-specific miRNAs were found to be up-regulated in response to ischemia/reperfusion injury in the rat heart [5] and in the myocardial infarction rat model [6]. Subsequent studies demonstrated that these miRNAs are involved in apoptotic cell death induced by cardiac ischemia through the post-transcriptional repression of the anti-apoptotic proteins B-cell lymphoma-2 and insulin-like growth factors [5], [6]. Conversely, other miRNAs were discovered to produce opposing effects on apoptosis by targeting heat shock protein 60, heat shock protein 70 and caspase-9 in cardiomyocytes [7].

Very recently, a series of studies have demonstrated that miRNAs are remarkably stable and can be readily quantified in human or animal serum and plasma. More importantly, circulating miRNAs demonstrate significant dynamic change in some pathological conditions and can partly reflect tissue damage [8], [9]. These discoveries have laid the ground work for potentially using circulating miRNAs as non-invasive biomarkers of cardiac disease. Towards that aim, efforts have been made to search for appropriate circulating miRNAs that could be novel and useful biomarkers for CAD [10], [11]. Thus far, at least 40 non-invasive, blood-based studies have examined miRNA expression profiles in order to identify miRNA biomarkers for the diagnosis of CAD. Most of them have focused on miRNAs in the serum or plasma of acute myocardial infarction patients and discovered some cardio-specific miRNAs including miR-1, miR-133 and miR-208 in acute myocardial infarction patients which were significantly increased compared to control subjects. These findings suggest that these circulating miRNAs might be potential diagnostic markers for acute myocardial infarction [10], [11]. These studies have shown promising results and have suggested that circulating miRNAs represent interesting candidates that could potentially serve as non-invasive biomarkers of cardiovascular disease. However, most of the results are based on a limited number of patients and few specific miRNAs, which may partially explain some the discrepancies observed. Moreover, these studies failed to explore the potential diagnostic value of circulating miRNAs in screening ACAD patients. We therefore hypothesized that circulating miRNAs signatures may be used as a novel tool to stratify ACAD. In the present study, using blood samples obtained from ACAD patients, we aimed to find a cluster of miRNAs that could be used as non-invasive and new risk-markers for the diagnosis of ACAD.

## Materials and Methods

### Ethics Statement

The present study was approved by the ethics committee board of Jinling hospital. All the patients and healthy volunteers from whom serum samples were obtained provided written informed consent prior to the study.

### Study population

A total of 127 consecutive patients who were admitted to the department of cardiology, Jinling Hospital (Nanjing, China) between November 2011 and June 2012 were enrolled in this study. The inclusion criteria for the ACAD patient population were as follows: 1) ≤1–2 of the 5 characteristics based on the Duke Clinical Score [12], including none; 2) normal level of the myocardial enzyme, high-sensitivity troponin I (hsTnI), and other biochemical indicators; 3) at least one coronary artery with significant stenosis; 4) uncommon reasons for referral such as CAD risk factors, atrial fibrillation, and pre-operative evaluation (*e.g.* Electrocardiogram, Treadmill Test, Ultrasonic cardiograph, Myocardial perfusion imaging *etc.*), other than chest pain or dyspnea. The exclusion criteria for all subjects were a previous history of cardiac disease (e.g., myocardial infarction, heart failure, cardiac arrhythmias, pacing, and cardiomyopathy), known history of leukopenia, thrombocytopenia, malignancy, severe hepatic or renal dysfunction, surgery or skeletal muscle damage, and evidence for inflammatory disease. Each patient was interviewed to collect demographic characteristics and medical history and underwent coronary angiography. Coronary angiograms were obligatorily combined with intravascular ultrasound and were evaluated independently by two cardiologists who made a visual estimation of luminal narrowing in multiple segments. According to the American Heart Association/American College of Cardiology classification of the coronary tree, a significant atherosclerotic lesion is defined as at least one major epicardial vessel with >50% stenosis as assessed by quantitative coronary angiography. Patient characteristics are summarized in **Table 1**. 45 normal controls were randomly selected from healthy individuals who had contemporaneously visited Jinling Hospital for a routine physical examination who were found to be normal on physical examination, electrocardiographic evaluation and laboratory tests without evidence of diseases such as hyperlipemia, hypertension, acute cardiovascular or cerebrovascular disease, diabetes mellitus, respiratory tract infections or any clinical evidence of atherosclerosis.

**Table 1:** Demographic and clinical features of the Atypical coronary artery disease (ACAD) patients and controls in the training set and validation set^a^.

	Training set			Validation set		
	Control	ACAD	*p*-value	Control	ACAD	*p*-value^b^
	(n = 10)	(n = 30)		(n = 34)	(n = 92)	
**Age (years)**	59.0±13.4	67.0±10.7	0.589	59.4±13.1	65.2±10.5	0.122
**Male sex**, n (%)	6 (60)	12 (40)	0.463	15 (44.1)	53 (57.6)	0.177
**Clinical features**						
Height (cm)	170.33±7.24	168.65±5.03	0.422	167.88±6.21	169±5.89	0.876
Body weight (kg)	71.67±12.06	71.23±10.82	0.856	70.23±10.82	71.88±11.56	0.556
Body mass index	25.53±4.02	24.99±2.56	0.453	24.82±2.72	25.83±1.48	0.466
**Blood parameter**						
AST (IU/L)	39.27±20.83	30.80±19.83	0.444	33.30±13.83	27.92±26.71	0.331
ALT (IU/L)	29.55±16.92	26.72±9.68	0.442	21.41±4.95	26.57±21.08	0.096
CK (IU/L)	79.00±85.44	87.33±58.34	0.772	68.48±24.50	85.23±42.59	0.063
CK-MB (IU/L)	15.55±11.84	10.67±3.09	0.205	12.00±7.44	11.58±4.08	0.748
Serum creatinine (mg/dl)	78.82±10.35	79.60±26.22	0.924	73.02±14.79	76.11±10.36	0.335
LDH (IU/L)	179.45±14.86	185.53±47.37	0.547	169.30±53.13	186.22±25.12	0.122
cTnI (ng/ml)	0.01±0.01	0.13±0.38	0.302	0.02±0.02	0.08±0.34	0.386
Platelets (×10^3^/µL)	180.91±72.04	184.87±60.42	0.861	192.96±51.97	171.60±50.99	0.082
RBC (×10^4^/µL)	4.59±0.441	4.40±0.485	0.272	4.52±0.38	4.41±0.54	0.273
WBC (µL)	6.19±2.548	6.21±1.543	0.779	6.18±2.01	6.68±1.51	0.027
Ca^2+^ (mmol/L)	2.22±0.06	2.28±0.32	0.512	2.16±0.10	2.12±0.11	0.091
Cl^−^ (mmol/L)	103.00±3.29	105.03±8.21	0.45	104.93±3.20	106.96±2.31	0.05
K^+^ (mmol/L)	4.00±0.31	3.99±0.47	0.966	4.17±0.40	3.92±0.33	0.004
Na^+^ (mmol/L)	142.00±2.61	141.73±4.23	0.846	141.85±2.66	143.75±2.72	0.004
Cholesterol (mmol/L)	4.23±0.79	4.54±1.57	0.306	4.36±1.07	4.69±1.07	0.204
Total glycerin (mmol/L)	1.28±0.21	1.56±0.69	0.056	1.33±0.25	1.44±0.22	0.51
HDL (mmol/L)	1.01±0.24	0.89±0.21	0.167	1.13±0.70	1.27±0.99	0.67
LDL (mmol/L)	2.60±0.68	3.18±1.41	0.218	2.58±0.93	2.99±0.98	0.071
**Other disease**						
Hypertension, n (%)	4 (40)	19 (63.3)	0.356	16 (47.1)	62 (67.4)	0.512
Diabetes mellitus, n (%)	4 (40)	16 (53.3)	0.465	19 (55.9)	55 (59.8)	0.693
Hyperlipidaemia, n (%)	3 (30)	16 (53.3)	0.360	9 (26.5)	59 (64.1)	0.36
Cerebral vascular event,n (%)	0 (0)	5 (16.7)	0.408	6 (17.6)	11 (12.0)	0.592
Arrhythmia, n (%)	4 (40)	9 (30)	0.845	9 (26.5)	36 (39.1)	0.268
**Smoking status**,n (%)	1 (10)	11 (36.7)	0.231	17 (50)	35 (38.0)	0.226
**Alcohol consumption**,n (%)	1 (10)	8 (26.7)	0.512	12 (35.3)	32 (34.8)	0.957

a Age data are presented as the mean (SD).

b Student-t test.

### Sample collection and processing

A total of 5 mL of venous blood was obtained by venipuncture from each donor after a 12 h overnight fast. The sample was immediately centrifuged at 1,500 g for 10 min at room temperature, followed by a 10-min high-speed centrifugation at 10,000 g at 4°C to completely remove cell debris. The samples were stored at −80°C until analysis.

### RNA extraction

For the TaqMan Low Density Array, equal volumes of serum from 35 ACAD patients and 20 normal subjects were pooled separately to form patient and control sample pools (total volume for each pool is ∼10 mL). RNA was extracted from each pooled sample according to a previously described protocol [13]. Briefly, 10 mL pooled serum was mixed with Trizol Reagent (Invitrogen, Carlsbad, Calif., USA) with a 1∶2 ratio and the samples were homogenized by vortexing vigorously for 1 min. After incubation in room temperature for 10 min, the mixture was centrifuged at 4°C for 15 min. After phase separation, the aqueous layer was transferred to a new tube and mixed with 1.5 volumes of isopropyl alcohol. This solution was stored at −20°C for 1 h. The RNA pellet was collected by centrifugation at 10,000 g for 20 min at 4°C. The resulting RNA pellet was washed once with 75% ethanol and dried for 10 min at room temperature. Finally, the pellet was dissolved in 20 μL of RNase-free water and stored at −80°C until further analysis.

For the RT-qPCR assay, an amount of 100 µL of serum was mixed with 200 µL of acid phenol, 200 µL of chloroform, and 300 µL of RNase-free water. The mixture was vortex-mixed vigorously and centrifuged at room temperature for 15 min. After phase separation, the aqueous layer was mixed with 1.5 volumes of isopropyl alcohol and 0.1 volumes of 3 mol/L sodium acetate (pH 5.3). This solution was stored at −20°C for 1 h. The RNA pellet was collected by centrifugation at 16,000 g for 20 min at 4°C. The resulting RNA pellet was washed once with 75% ethanol and dried for 10 min at room temperature. Finally, the pellet was dissolved in 20 µL of RNase-free water and stored at −80°C until further analysis.

### TaqMan low-density array and RT-qPCR analysis

TaqMan Human MicroRNA A and B Arrays, version 3.0 (Applied Biosystems, Foster City, CA, USA), were used for miRNA expression screening of 754 serum miRNAs on an ABI PRISM 7900HT instrument. To increase the sensitivity of the TaqMan Low Density Array, a pre-amplification was performed after the reverse transcription. All reactions were performed as specified in the manufacturer's protocol. The serum miRNA expression levels in individual samples were determined by a TaqMan probe-based RT-qPCR on a 7300 Real-Time PCR Sequence Detection System (Applied Biosystems). Because U6 and 5S rRNA are degraded in serum samples and the lack of a consensus housekeeping miRNA for the RT-qPCR analysis of serum miRNAs, miRNA expression was normalized to serum volume. Briefly, 2 µL of total RNA was reverse-transcribed to cDNA using the AMV reverse transcriptase (TaKaRa, Dalian, China) for synthesis of cDNA, the reaction mixtures were incubated at 16°C for 30 min, at 42°C for 30 min, at 85°C for 5 min, and then held at 4°C. Real-time PCR was performed using a TaqMan PCR kit according to the manufacturer’s instructions with a minor modification as described in a previous study. The Real-Time PCR cycles consisted of pre-denaturation at 1 cycle of 95°C for 5 min, and 40 cycles of 95°C for 15 sec and 60°C for 1 min. All reactions, including no-template controls, were performed in triplicate. The resulting Cq values were determined using fixed threshold settings. We assessed the detection limits of the RT-qPCR assay, dynamic range and calculated the absolute concentration of target miRNAs based on a calibration curve developed by synthetic miRNA oligonucleotides with known concentrations.

### miRNA Target gene prediction

Targets of the five altered miRNAs were created by combining predicted results from the public database including TargetScan, miRanda, and PicTar. Potential targets were selected based on gene function, the number of predicted target sites, and target prediction by multiple algorithms. The NCBI DAVID server (http://david.abcc.ncifcrf.gov/tools.jsp) was used to provide further information about the corresponding genes’ functions.

### Statistical analysis

Statistical analysis was performed with SPSS 16.0 software (SPSS, Inc., Chicago, USA). The data are presented as the means ± SEM for serum miRNA levels or means ± SD for other variables. Non-parametric Mann-Whitney tests were used to compare the differences in serum miRNA expression levels between the ACAD group and healthy controls group. Student’s t-test was used to compare the differences in other variables between the two groups. A *p*-value<0.05 was considered statistically significant. The receiver operating characteristic (ROC) curve was generated and the area under the curve (AUC) was calculated to evaluate the specificity and sensitivity of ACAD prediction for each serum miRNA. Risk score analysis was performed to evaluate the associations between ACAD and the expression levels of the serum miRNAs as previously described [14]. In brief, the risk score of the upregulated miRNA, denoted as “s”, was set to 1 if the expression level was greater than the upper 95% reference interval for the corresponding miRNAs level in controls and was set to 0 in all other cases. For the downregulated miRNAs, the “s” was set to 1 if the expression level was lower than the lower 5% reference interval for the corresponding miRNAs level in controls and was set to 0 in all other cases. When taking into account the correlation of each miRNA with ACAD risk, each patient was assigned a risk score function (RSF) according to a linear combination of the expression level of the miRNA. The RSF for sample i using the information from the five miRNAs was: rsf_i_ = ∑^5^_j-1_W_j_.s_ij_.

In the above equation, s_ij_ is the risk score for miRNA j on sample i, and W_j_ is the weight of the risk score of miRNA j. To determine the Ws, five univariate logistic regression models were fitted using the disease status with each of the risk scores. The regression coefficient of each risk score was used as the weight to indicate the contribution of each miRNA to the RSF. The frequency table and ROC curves were then used to evaluate the diagnostic effects of the profiling and to find the appropriate cutoff point.

## Results

### Baseline clinical characteristics of the study population

We recruited 166 participants including 127 ACAD patients (69 men and 58 women; mean age, 66.8±10.3 years) and 54 healthy volunteers (29 men and 25 women, mean age 61.3±14.5 years). All ACAD patients were selected on the basis of clinical parameters (e.g. Signs and symptoms, history, exam and lab value, *etc.*) combined with angiographic documentation. The demographics and clinical features of the patients enrolled in training set and validation set are listed in **Table 1**. Healthy controls were recruited from a large pool of individuals seeking a routine health check-up at the Healthy Physical Examination Centre of Jinling Hospital. People without evidence of any disease were selected as control subjects. Control subjects were matched to the patients by age, sex and ethnicity.

### TaqMan low-density array analysis of serum miRNA in ACAD patients

**Figure 1:**
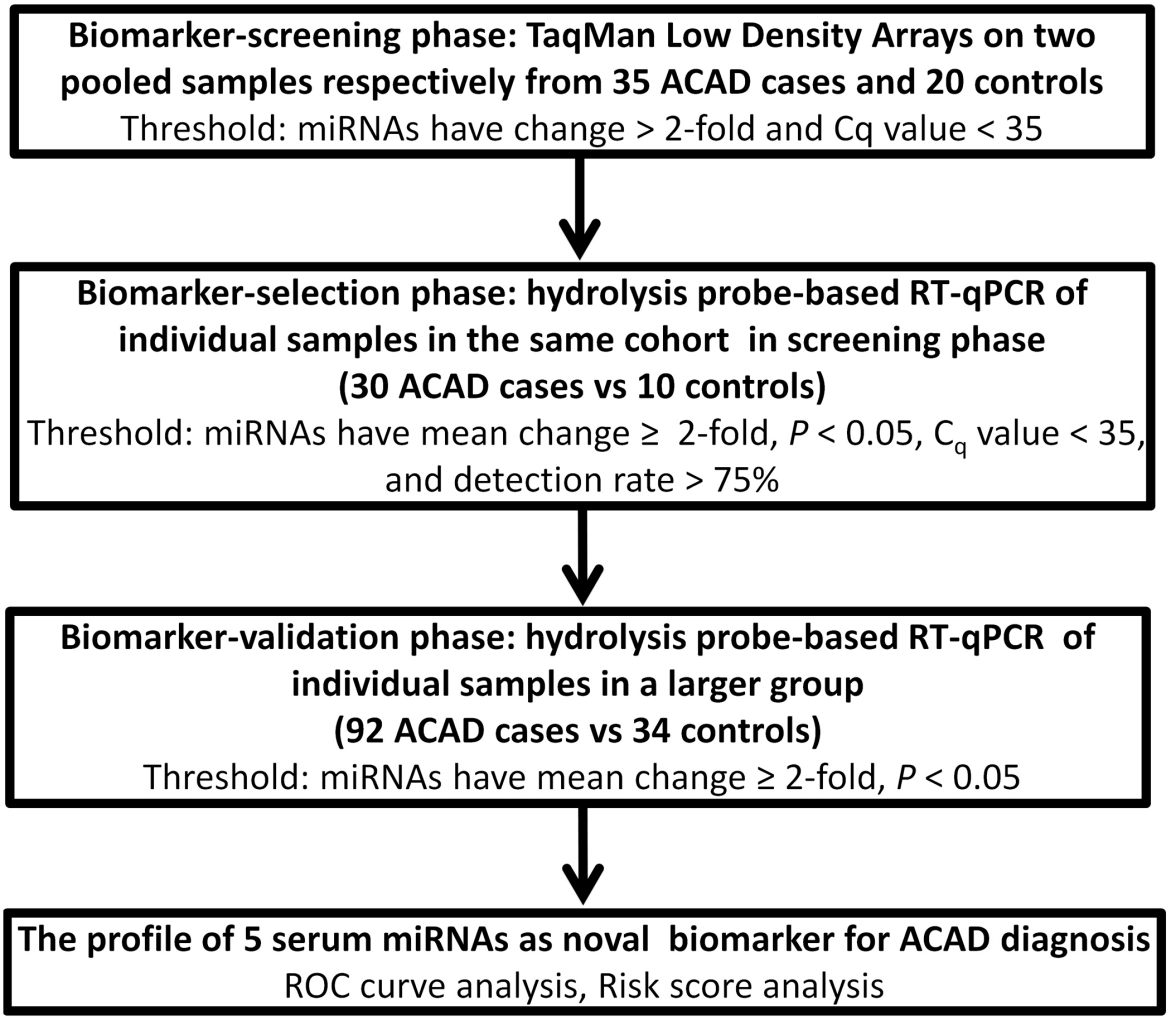
Overview of the experimental design.

A two-phase case-control study was designed to assess serum miRNAs as a surrogate marker for ACAD (**Figure 1**). We first performed a TaqMan low-density array analysis to screen and select candidate miRNAs that showed markedly alterations in pooled serum samples between ACAD patients and healthy controls. Of the 766 miRNAs scanned, 315 and 286 miRNAs could be detected in the sera of ACAD patients and healthy controls, respectively. To further identify miRNAs with differential expression levels between the two groups, we used two criteria: (1) Cq values <30 and (2) miRNA levels showed at least 100-fold difference. These two criteria yielded a list of 44 differentially expressed miRNAs, 22 of which were up-regulated and 22 down-regulated in ACAD patients compared with healthy controls (**Table S1**).

### Confirmation of the altered serum miRNAs levels in ACAD patients by RT-qPCR analysis

**Figure 2:**
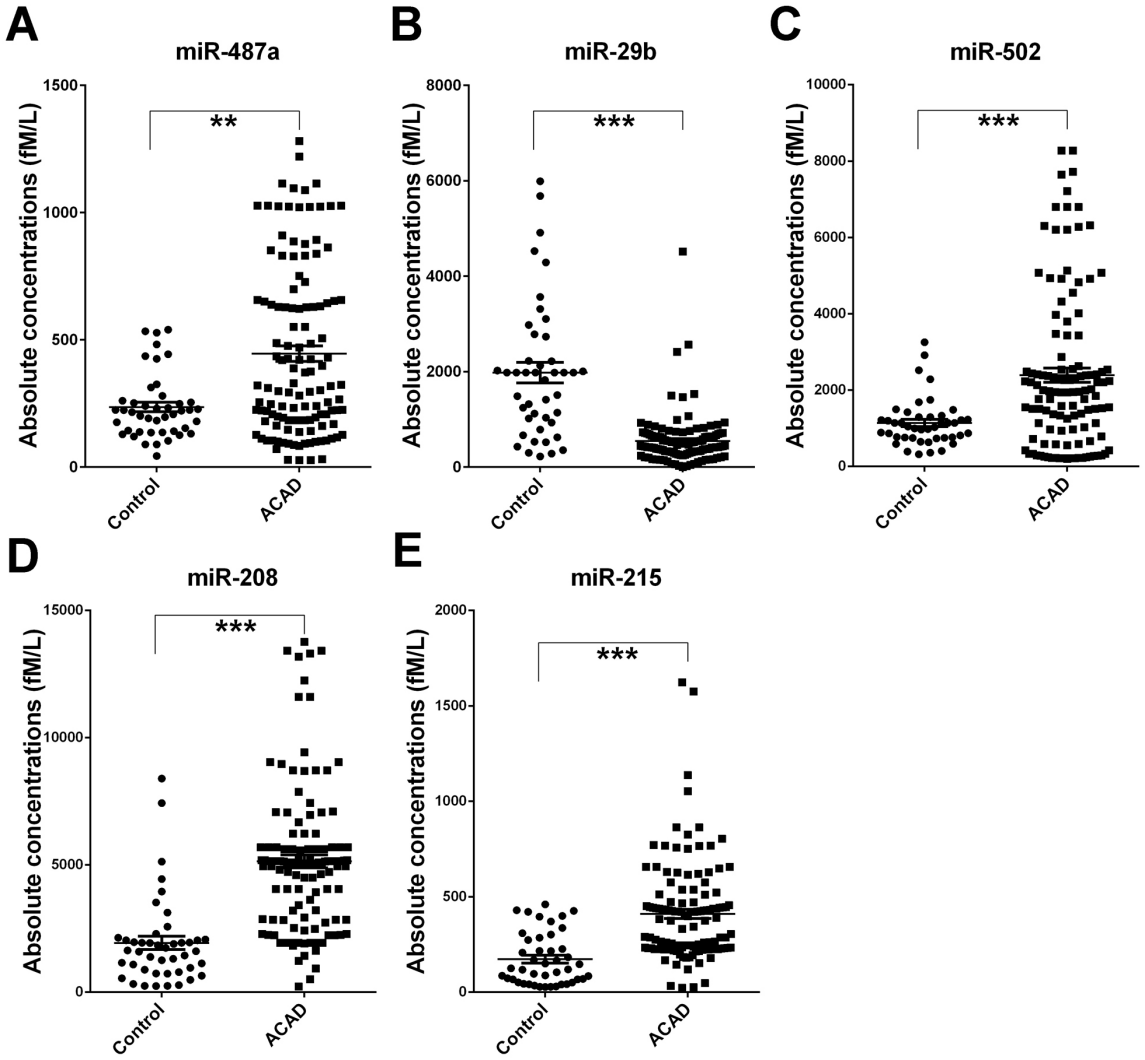
Differential expression of the five miRNAs (A) miR-487a, (B) miR-29b, (C) miR-502, (D) miR-208 and (E) miR-215 in the serum between Atypical Coronary Artery Disease (ACAD) cases and controls. The expression levels of the five miRNAs are detected by RT-qPCR individually. Serum levels of the five miRNAs were measured in 122 AD cases and in 44 controls of training set and validation set using a hydrolysis probe-based RT-qPCR assay. Cq values were converted to absolute values based on the standard curves. Each point represents the mean of triplicate samples. Each *p*-value was derived from a nonparametric Mann–Whitney *U*-test. **p<0.01; ***p<0.001.

We next employed a TaqMan probe-based RT-qPCR assay to confirm the expression of the candidate miRNAs selected from the TaqMan low-density array analysis. The serum samples were arranged in two sets including a biomarker screening set (training set) and biomarker verification set (validation set). In the training set, miRNAs were detected in a set of individual serum samples including 30 ACAD patients and 10 normal controls which from the same cohort of TaqMan low-density array screening phase. Only those miRNAs with a mean fold-change ≥2.0 and a *P*-value <0.05 were chosen for further analysis. Moreover, miRNAs with a Cq value >35 and a detection rate <75% in either the ACAD or control group were excluded. Based on this criteria, four miRNAs including miR-487a, miR-502, miR-208 and miR-215 were markedly increased in serum from ACAD patients compared to control subjects (*p*<0.05), whereas miR-29b were significantly lower than in control subjects (*p*<0.001) (**Table 2**). Expression levels of the five altered miRNAs were then chosen from the training set and further confirmed in a large cohort samples set (validation set) composed of 92 ACAD patients and 34 age-gender matched controls. Consistent with the results from the training set, serum levels of miR-487a, miR-502, miR-208 and miR-215 were significantly higher in the ACAD patients compared to the control subjects, and miR-29b was significantly decreased with a similar trend to the previous results (**Table 2**). The differences in concentration for these seven miRNAs in all ACAD patients and the control individuals enrolled in the training and validation sets are shown in **Figure 2**.

**Table 2:** Differentially-expressed miRNAs in Atypical coronary artery disease (ACAD) serum samples compared to control samples in training set and validation set^a^.

	Training set			Validation set		
miRNA	Control	ACAD	*P* value^b^	Control	ACAD	*P* value^b^
	(n = 10)	(n = 30)		(n = 34)	(n = 92)	
miR-487a	184.27±23.54	400.95±41.28	0.002	251.31±23.03	460.59±37.71	0.027
miR-29b	2290.91±410.84	635.31±145.71	<0.001	1890.08±251.39	520.97±42.02	<0.001
miR-502	1229.97±232.18	3186.68±368.78	<0.001	1116.99±102.41	2772.15±412.40	0.016
miR-208	1551.19±167.21	3839.48±452.85	<0.001	2051.02±330.05	5574.02±293.53	<0.001
miR-215	86.76±24.00	385.73±37.45	<0.001	198.80±23.97	418.96±28.89	<0.001

a The absolute concentrations of miRNAs are presented as mean ± SEM (fM/L).

b Mann-Whitney unpaired test for rank sum.

### Receiver Operating Characteristic Analysis

**Figure 3:**
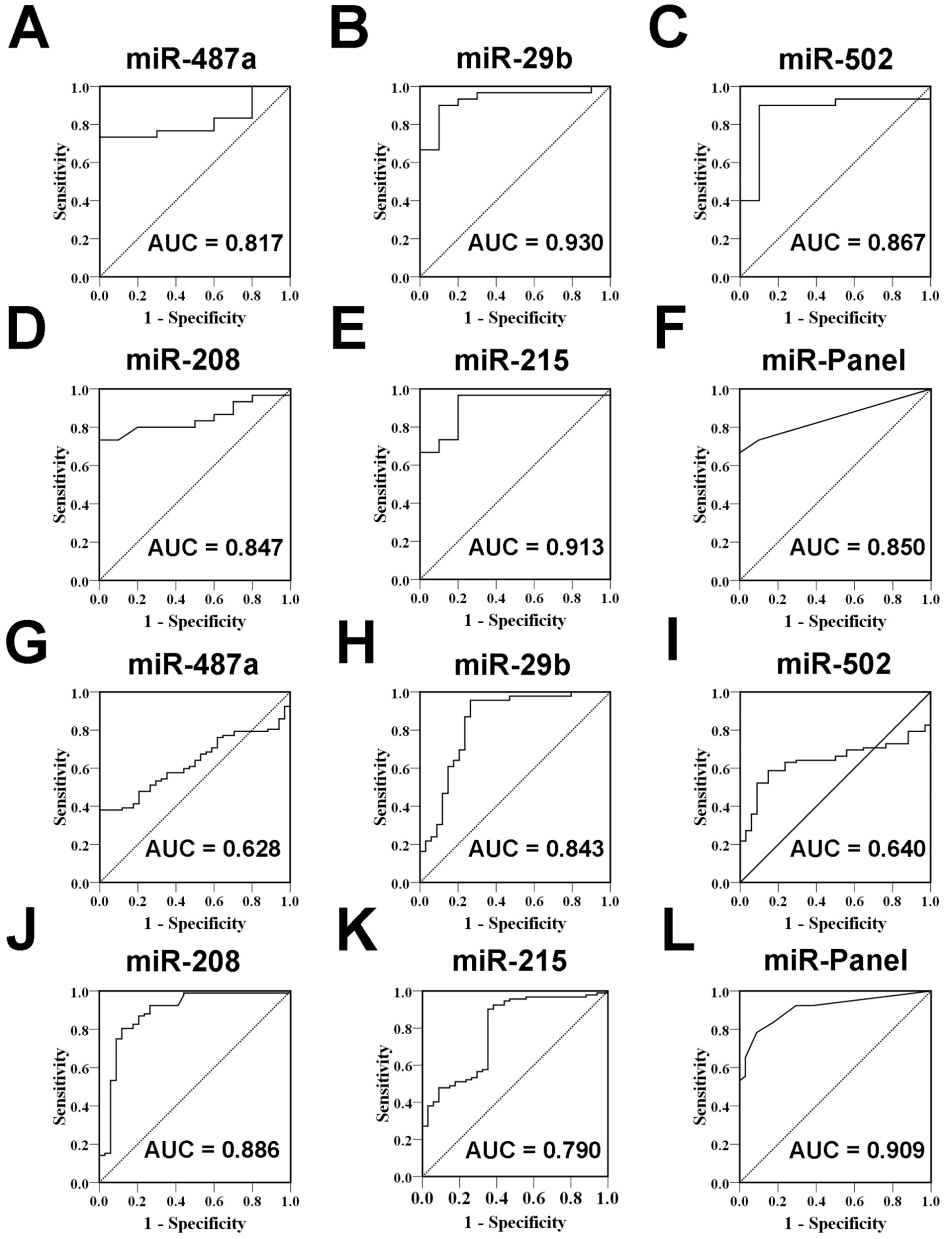
The receiver operating characteristic (ROC) curve analysis for discriminative ability between the Atypical Coronary Artery Disease (ACAD) cases and noncancer controls by the 5 miRNAs and their panel. ROC curves for the ability of the the 5 miRNAs and their panel to differentiate the 30 ACAD cases from the 10 controls in the training set (A–F), 92 ACAD cases from the 30 controls in the validation set (G–L).

To evaluate the usefulness of the five altered circulating miRNA as potential biomarkers for ACAD, we conducted a ROC analysis. When a comparison was made between ACAD patients and healthy controls of biomarker training set, the AUC for these miRNAs ranged from 0.817 to 0.930 (**Figure 3**). To determine the diagnostic value of the combination of the five miRNAs, we performed a risk score analysis on the data set and used it to predict ACAD case and control status. The frequency table and the ROC curves were then used to evaluate the diagnostic effect of the five-miRNA panel. When using the optimal cut off value, where the sum of sensitivity and specificity was maximal, the diagnostic sensitivity and specificity of the five miRNA–based markers for ACAD detection were 66.7% and 100%, respectively, and the AUCs were 0.850 (95% CI, 0.734–0.966, *P*<0.001) (**Figure 3**). The diagnostic values of these five miRNAs were further evaluated in the biomarker validation set. As shown in the Figure 3, the AUCs for these five miRNAs ranged from 0.628 to 0.886. When using the same risk score formula to calculate the risk score of samples from the verification set and constructed ROC curves using these RSFs to estimate the diagnostic sensitivity and specificity of the 5-miRNA–based biomarker. The diagnostic sensitivity and specificity of the five-miRNA panel for ACAD detection in the validation set were 83.7% and 82.4%, respectively. Furthermore, the ROC curve for the panel revealed a pronounced diagnostic accuracy, evidenced by the AUC of 0.909 (95% CI, 0.858–0.960, P<0.0001), which was much better than that for five individual miRNA (**Figure 3**). Moreover, we also investigated the five miRNAs and their different panels in all the ACAD cases and controls of training set and validation set, and found that both panels of the five-serum miRNAs (miR-487a, miR-502, miR-208, miR-215 and miR-29b) and other different panels as well as individual miRNA (**Table S2** and **Table S3**) could reliably discriminate ACAD from controls, and the panel of five-serum miRNAs had a significant higher AUC when compared with the other panels, which indicated that the five-miRNA panel is really a comprehensive and specific indicator than other panels for ACAD.

To further evaluate the diagnostic value of the 5-miRNA profiling system, we performed a risk score formula to calculate the RSF for ACAD patients and control samples according to above description and previous study [14], [15]. Based on their risk scores and at a set cutoff, samples could be divided into a high-risk group representing the predicted ACAD cases, or a low risk group representing the predicted controls. When using the optimal cutoff value (RSF = 2.1305) at which the sum of the sensitivity and specificity was maximal, the specificity and sensitivity were 78.7% and 86.4%, respectively. At this cutoff, 38 of the 44 controls had RSF values <2.1305, while 95 of the 122 ACAD samples had a risk score >2.1305 (**Table 3**).

**Table 3:** Risk score analysis of ACAD cases and control donors.

		0∼2.1305	2.1305∼10.478	PPV^a^	NPV^b^
Training set	Control	10	0	100%	50%
	ACAD	10	20		
Validation set	Control	28	6	92.60%	62.20%
	ACAD	17	75		

a PPV, positive predictive value;

b NPV, negative predictive value.

We next performed an ROC analysis for traditional CAD protein marker plasma hsTnI between all ACAD patients and normal subjects in order to further weigh the usefulness of the selected miRNAs. The AUC for plasma hsTnI was 0.627 (95% confidence interval; CI = 0.536–0.718), significantly lower than AUC for the five miRNA and their panel (**Table S2 and Table S3**). These data implied that the five-serum miRNA signature could represent a suitable biomarker that allowed for efficient differentiation of ACAD patients from other subjects.

### Prediction of the five altered miRNAs mediated functional gene regulation

To explore the possible regulatory role of miR-487a, miR-502, miR-208, miR-215 and miR-29b in ACAD, gene targets were predicted by using miRNA target prediction databases, namely, Target Scan, miRanda and PicTar. The predicted targets were then compared with a list of upregulated and downregulated genes identified in other earlier studies. Functional classification of the target genes was carried out with gene ontology analysis using the DAVID tool. Our analysis showed that some genes such as transforming growth factor-β activated kinase-1 binding (TAB) protein 3 and catenin-beta interacting protein 1 targeted by miR-487a and miR-215 were involved in the inflammatory process. Among the predicted genes (such as vascular endothelial growth factor A, microfibrillar-associated protein 3, Kv channel interacting protein 1 and TNF receptor-associated factor 3) that were targeted by miR-361-5p, vascular endothelial growth factor A was found to be a key point in a signaling pathway for vasculogenesis and endothelial cell growth, cell migration, cell apoptosis and arteriosclerosis. Consistent with the earlier published articles, miR-208 and miR-29b apparently play a role in the process of vascular or myocardial remodeling.

## Discussion

At present, more than 40 studies have revealed alterations of miRNAs in serum or plasma in cardiovascular disease. These observations have led to rapid progress in the use of these small molecules as biomarkers for cardiovascular disease. However, those studies are mainly focused on miRNAs that are specifically expressed in skeletal muscle or cardiac tissues, and most of the selected candidate miRNA markers are only for acute myocardial infarction and CAD. This led us to perform a comprehensive analysis of the dynamic changes and diagnostic value of serum miRNAs in ACAD patients. To our knowledge, no previous studies have been conducted on circulating miRNAs in large cohorts of ACAD patients. In the current study, using a genome-wide TaqMan low-density array technology to determine the whole serum miRNAs expression levels of ACAD patients and normal controls followed by RT-qPCR confirmation in individual samples, we successfully identified a novel five miRNAs signature that included miR-487a, miR-502, miR-208, miR-215 and miR-29b and was markedly altered in the serum of ACAD patients. Further ROC curve analysis indicated that the AUC as well as the sensitivity and specificity of the selected miRNAs were better than the traditional protein marker hsTnI. We therefore conclude that these five circulating miRNAs may serve as independent biomarkers for the diagnosis of ACAD.

ACAD is usually referred to as atypical angina pectoris and silent myocardial ischemia, which could lead to a delay in proper evaluation or management. This description suggests that symptomatology is a less specific, and possibly less sensitive in defining myocardial injury. In light of the limitation of routine lab techniques [16], innovative and reliable biomarkers for the detection of ACAD are urgently needed. Though extraordinary efforts have been directed towards determining the molecular and pathological characteristics of this disease in order to develop novel diagnostic and therapeutic strategies, none have been validated for use in the clinical setting.

Recent studies have observed that miRNAs are stable when present in the circulation, and can be readily quantified by RT-qPCR technology. More importantly, unique serum miRNA expression profiles have been suggested for various diseases including cardiovascular disease and may serve as finger prints for their detection [8], [9]. For some time, peripheral blood has been attracting increasing attention from the scientific community for its use as a source for detecting clinical biomarkers of cardiovascular disease because it can be easily obtained in the clinical setting. Because miRNAs have been found to be stably expressed in blood and can be readily quantified by RT-qPCR, studies have highlighted the crucial role of serum miRNAs in diagnosing cardiovascular diseases such as acute myocardial infarction, CAD and ischemia. In our study, significant differences in the expression of 5 novel miRNAs were observed between the ACAD cases and controls. Of the five miRNAs, miR-208 has also been reported in multiple studies as being differentially expressed between normal and patient group. The biological basis for alterations of miRNAs in ACAD remains unclear. It is possible that it involves, at least to some degree, the body’s systemic response and/or genetic susceptibility to CAD.

The identification of miRNAs’ targets is crucial for elucidating their function. However, due to the complexity of the miRNA–target interactions, this step has proven computationally difficult. At present, several target prediction algorithms have been developed, but they show a poor overlap between their outputs suggesting that there are a number of false-negatives and as well as false-positive predictions [17]. The mechanisms responsible for ACAD are not well understood, and individual differences in pain threshold may only partially explain the variability in pain perception [18]–[20]. Pain perception may result from microenvironmental balances between proinflammatory cytokines (including interleukin-1β, tumor necrosis factor-α, interleukin-6, and interferon-γ) and anti-inflammatory cytokines (including interleukin-4, interleukin-10) [21], [22]. The proinflammatory activation seems to intensify nociception, whereas Th2 lymphocyte production seems to abolish the pain perception [23]. The predicted targets of most miRNAs uncovered in our study pointed towards a significant role in local inflammation. Therefore, we suspected that the miRNAs were involved in the imbalance between pro-inflammatory and anti-inflammatory cytokines and played a role in the atherosclerosis of ACAD. Of the five selected serum miRNAs in the present study, miR-215 has been reported to promote β-catenin activation and upregulate α-SMA and fibronectin expression in Transforming growth factor-β1 treated mouse mesangial cell by targeting catenin-beta interacting protein 1. All of these genes were reported to be participated in neointimal lesion formation [24]. Since neointimal lesions often occur at the site of subclinical atherosclerosis, we presumed subclinical atherosclerosis to be one possible contributor to silent myocardial ischemia. Furthermore, altered miR-215 could stimulate subclinical atherosclerosis via neointimal lesion formation. TGF-β has been found to be an atheroprotective factor in arteriosclerosis [25], and the TGF-β/Smad pathway can suppress NF-kB activation-induced inflammation by blocking TAB2 or TAB3 [26]. Our bioinformatics analysis showed that TAB3 is a potential target of miR-487a. This result in combination with existing reports led us to speculate that miR-487a may be involved in the occurrence of atherosclerosis by regulating TAB3 expression. Taken together, the above evidences may fuel the notion that miRNAs are associated with the TGF-β cascade and may be a potential therapeutic target for ACAD. Of the five selected miRNAs, miR-29b and miR-208 have been shown to be useful biomarkers for myocardial injury [27]. In our study, we found that miR-29b was significantly decreased in silent myocardial ischemia patients. The precise mechanism by which miR-29b participates in myocardial ischemia is still unclear; however, the predicted targets of miR-29b, including 20 collagens and several extracellular matrix genes, may explain the role of this miRNA in myocardial ischemia [28]. MiR-208 has been associated with cardiac remodeling [29] and elevated plasma miR-208 has been identified as one of the most promising biomarkers for myocardial injury [30], [31]. Our results showed that levels of miR-208 were significantly elevated in ACAD patients when compared to normal controls. Intriguingly, a recent study reported that levels of miR-208 were much higher in angina pectoris cases than in acute myocardial ischemia cases [32], implying that miR-208 could be used to monitor the early stages of myocardial injury. Our ROC analysis showed that the sensitivity and specificity of miR-208 for ACAD diagnosing is much higher than that of hsTnI. Based on these results, we concluded that serum miR-208 is more valuable than Troponin I in diagnosing ACAD. For miR-502, which is often expressed in myogenic differentiation [33], has been regarded as a “myogenic miRNA”. One group suggested that miR-502 could suppress the autophagy process and play an atheroprotective role by directly targeting RAB1B and AP2B1. While reduced miR-502 could then become the potential section that accounted for the inconspicuous myocardial insult [34], [35], the exact mechanism underlying miR-502's role in ACAD requires further study.

In summary, we successfully identified five miRNAs that showed different expression levels in ACAD patient serum and controls. Moreover, the five miRNAs had a high diagnostic value in identifying coronary artery disease, particularly with atypical presentation. The bioinformatics prediction indicated all five miRNAs may be involved in the pathogenesis of CAD. As a novel invasive biomarker, the established miRNAs and their panel could potentially decrease the number of individuals with under-recognized coronary lesions and help to avoid misdiagnosis and excessive medical treatment.

## Supporting Information

Table S1 **Differentially-expressed miRNAs in ACAD serum samples compared to normal controls determined by TaqMan Low Density Assay.**
(DOCX)

Table S2 **ROC curves and the corresponding AUCs of the five selected miRNAs for all the ACAD patients and controls in training set and validation set.**
(DOCX)

Table S3 **ROC curves and the corresponding AUCs of different serum-miRNA panels for all the ACAD patients and controls in training set and validation set.**
(DOCX)
