# Supplementary material for: Altered Serum MicroRNAs as Novel Diagnostic Biomarkers for Atypical Coronary Artery Disease
Source: PLoS One. 2014 Sep 8;9(9):e107012. doi: 10.1371/journal.pone.0107012 (PMC4157840; doi:10.1371/journal.pone.0107012)
Supplement: Table S1 — Differentially-expressed miRNAs in ACAD serum samples compared to normal controls determined by TaqMan Low Density Assay. (DOCX) [file pone.0107012.s001.docx]

**Table S1 Differentially-expressed miRNAs in ACAD serum samples compared to normal controls determined by TaqMan low density array.**

| miRNA | ACAD | | Control | | Delta Delta Ct | Fold change |
| --- | --- | --- | --- | --- | --- | --- |
|  | Raw Ct | Delta Ct | Raw Ct | Delta Ct |  |  |
| miR-338-5p | 9.224622 | -7.43904 | 40 | 22.09068 | -29.52971067 | 775048132.1 |
| miR-502 | 19.95861 | 3.29495 | 40 | 22.09068 | -18.79572267 | 455068.0162 |
| miR-487a | 21.29777 | 4.63411 | 40 | 22.09068 | -17.45656467 | 179866.2109 |
| miR-208b | 21.67846 | 5.0148 | 40 | 22.09068 | -17.07587167 | 138149.5815 |
| let-7e | 23.07849 | 6.41483 | 40 | 22.09068 | -15.67584167 | 52347.8138 |
| miR-215 | 23.57895 | 6.91529 | 40 | 22.09068 | -15.17538667 | 37003.82194 |
| miR-339-5p | 24.0047 | 7.34104 | 40 | 22.09068 | -14.74963267 | 27547.47881 |
| miR-10b | 25.00255 | 8.33889 | 40 | 22.09068 | -13.75178267 | 13794.28125 |
| mmu-let-7d* | 25.9405 | 9.27684 | 40 | 22.09068 | -12.81383767 | 7200.281855 |
| miR-211 | 26.81732 | 10.1537 | 40 | 22.09068 | -11.93701667 | 3921.02919 |
| miR-210 | 26.96118 | 10.2975 | 40 | 22.09068 | -11.79315667 | 3548.901129 |
| miR-330 | 27.12407 | 10.4604 | 40 | 22.09068 | -11.63026167 | 3169.988006 |
| miR-501-3p | 28.00802 | 11.3444 | 40 | 22.09068 | -10.74630867 | 1717.755119 |
| miR-208 | 28.77921 | 12.1156 | 40 | 22.09068 | -9.975119667 | 1006.491775 |
| miR-130b* | 28.94288 | 12.2792 | 40 | 22.09068 | -9.811449667 | 898.546694 |
| miR-505 | 28.96876 | 12.3051 | 40 | 22.09068 | -9.785571667 | 882.572917 |
| miR-1183 | 15.12575 | -1.5379 | 25.852522 | 7.943197 | -9.481100667 | 714.6537758 |
| miR-148a | 26.94019 | 10.2765 | 37.26684 | 19.35752 | -9.080979667 | 541.5608606 |
| miR-1303 | 29.95359 | 13.2899 | 40 | 22.09068 | -8.800747667 | 445.9529405 |
| miR-126* | 24.95857 | 8.29491 | 33.99709 | 16.08777 | -7.792853667 | 221.7597409 |
| miR-370 | 26.03156 | 9.3679 | 35.014256 | 17.10493 | -7.737033667 | 213.3433995 |
| miR-193a-5p | 22.97606 | 6.3124 | 31.940182 | 14.03086 | -7.718454667 | 210.6135822 |
| miR-219 | 32.05353 | 15.3899 | 25.958487 | 8.049162 | 7.340710333 | 0.00616916 |
| miR-202 | 34.97829 | 18.3146 | 27.973698 | 10.06437 | 8.250263333 | 0.003284152 |
| miR-450b-5p | 34.96068 | 18.297 | 25.854204 | 7.944879 | 10.35214333 | 0.000765058 |
| miR-367 | 40 | 23.3363 | 29.922693 | 12.01337 | 11.32297433 | 0.000390342 |
| miR-650 | 40 | 23.3363 | 28.09012 | 10.1808 | 13.15554733 | 0.000109594 |
| miR-218-1* | 40 | 23.3363 | 27.918236 | 10.00891 | 13.32743133 | 9.72844E-05 |
| miR-335 | 30.03747 | 13.3738 | 17.77549 | -0.13383 | 13.50764333 | 8.58607E-05 |
| miR-645 | 40 | 23.3363 | 27.079544 | 9.170219 | 14.16612333 | 5.43966E-05 |
| miR-29b | 30.99183 | 14.3282 | 17.974533 | 0.065208 | 14.26296733 | 5.0865E-05 |
| miR-1247 | 40 | 23.3363 | 26.966091 | 9.056766 | 14.27957633 | 5.02828E-05 |
| miR-492 | 40 | 23.3363 | 26.85745 | 8.948125 | 14.38821733 | 4.66353E-05 |
| miR-199a | 40 | 23.3363 | 26.731348 | 8.822023 | 14.51431933 | 4.27321E-05 |
| miR-205 | 40 | 23.3363 | 25.829103 | 7.919778 | 15.41656433 | 2.2864E-05 |
| miR-299-3p | 40 | 23.3363 | 24.955502 | 7.046177 | 16.29016533 | 1.24788E-05 |
| miR-129* | 40 | 23.3363 | 24.651085 | 6.74176 | 16.59458233 | 1.01049E-05 |
| miR-130a* | 40 | 23.3363 | 23.933617 | 6.024292 | 17.31205033 | 6.14545E-06 |
| miR-412 | 40 | 23.3363 | 22.778875 | 4.86955 | 18.46679233 | 2.76021E-06 |
| miR-380-3p | 40 | 23.3363 | 22.02063 | 4.111305 | 19.22503733 | 1.63188E-06 |
| miR-193a-3p | 40 | 23.3363 | 18.847431 | 0.938106 | 22.39823633 | 1.80909E-07 |
| miR-504 | 40 | 23.3363 | 17.524006 | -0.38532 | 23.72166133 | 7.22884E-08 |
| miR-216a | 40 | 23.3363 | 16.933218 | -0.97611 | 24.31244933 | 4.7998E-08 |
| miR-198 | 40 | 23.3363 | 16.890045 | -1.01928 | 24.35562233 | 4.6583E-08 |
