# Supplementary material for: Altered Serum MicroRNAs as Novel Diagnostic Biomarkers for Atypical Coronary Artery Disease
Source: PLoS One. 2014 Sep 8;9(9):e107012. doi: 10.1371/journal.pone.0107012 (PMC4157840; doi:10.1371/journal.pone.0107012)
Supplement: Table S2 — ROC curves and the corresponding AUCs of the five selected miRNAs for all the ACAD patients and controls in training set and validation set. (DOCX) [file pone.0107012.s002.docx]

**Table S2 ROC curves and the corresponding AUCs of the five selected miRNAs for all the ACAD patients and controls in training set and validation set.**

| miRNA | Area | Std. Errora | Asymptotic Sig. | Asymptotic 95% Confidence Interval | |
| --- | --- | --- | --- | --- | --- |
|  |  |  |  | Lower Bound | Upper Bound |
| miR-487a | 0.670 | 0.041 | 0.001 | 0.590 | 0.750 |
| miR-29b | 0.867 | 0.037 | <0.0001 | 0.795 | 0.939 |
| miR-502 | 0.695 | 0.040 | <0.0001 | 0.617 | 0.774 |
| miR-208b | 0.876 | 0.033 | <0.0001 | 0.811 | 0.941 |
| miR-215 | 0.826 | 0.037 | <0.0001 | 0.754 | 0.898 |
| miR-Panel | 0.885 | 0.025 | <0.0001 | 0.836 | 0.935 |
| hsTnI | 0.627 | 0.046 | 0.012 | 0.536 | 0.718 |
