# Supplementary material for: Altered Serum MicroRNAs as Novel Diagnostic Biomarkers for Atypical Coronary Artery Disease
Source: PLoS One. 2014 Sep 8;9(9):e107012. doi: 10.1371/journal.pone.0107012 (PMC4157840; doi:10.1371/journal.pone.0107012)
Supplement: Table S3 — ROC curves and the corresponding AUCs of different serum-miRNA panels for all the ACAD patients and controls in training set and validation set. (DOCX) [file pone.0107012.s003.docx]

**Table S3 ROC curves and the corresponding AUCs of different serum-miRNA panels for all the ACAD patients and controls in training set and validation set.**

| miRNA panel | Area | Std. Errora | *P*-value | 95% Confidence Interval | |
| --- | --- | --- | --- | --- | --- |
|  |  |  |  | Lower Bound | Upper Bound |
| miR-487a/29b panel | 0.700 | 0.042 | < 0.001 | 0.618 | 0.781 |
| miR-487a/29b/502 panel | 0.758 | 0.037 | < 0.001 | 0.685 | 0.831 |
| miR-487a/29b/502/208 panel | 0.852 | 0.03 | < 0.001 | 0.794 | 0.910 |
| miR-487a/29b/502/208/215 panel | 0.885 | 0.025 | < 0.001 | 0.839 | 0.938 |
| miR-29b/208/215 panel | 0.839 | 0.031 | < 0.001 | 0.779 | 0.899 |
| hsTnI | 0.627 | 0.046 | 0.012 | 0.536 | 0.718 |
